# ADULT MALTREATMENT OUTCOMES OF A TARGETED CASE MANAGEMENT INTERVENTION FOR SELF-NEGLECT

**DOI:** 10.1093/geroni/igac059.1598

**Published:** 2022-12-20

**Authors:** Courtney Reynolds, Farida Ejaz, Miriam Rose

**Affiliations:** Benjamin Rose Institute on Aging, Cleveland, Ohio, United States; Benjamin Rose Institute on Aging, Cleveland, Ohio, United States; Benjamin Rose Institute on Aging, Cleveland, Ohio, United States

## Abstract

A longitudinal study to prevent self-neglect among at-risk, older (age 60+) or disabled (age 18+) healthcare patients was conducted collaboratively by a gerontological research institute, a health care system, and Texas Adult Protective Services (APS). Selected patients (n = 285) from 19 primary care clinics received targeted case management services for four months by a trained social worker. Their median age was 70, 72% were Hispanic/Latino, 79% had a high school education or less, and 73% had monthly income less than $1,361. A screening tool was used to identify adult maltreatment during baseline and posttest interviews. Consistent with national prevalence rates, 86% of participants at baseline, and 90% at posttest, reported they had not recently experienced abuse. In this sample, we found a significant decrease in mean scores on the screening tool from baseline (M = 0.24, SD = 0.63) to posttest (M = 0.14, SD = 0.48); t(241), p < 0.05. Six of the seven participants reported by project social workers to APS had at least one allegation of self-neglect. APS administrative data were also examined and included another 10 study participants reported to APS during the study. In total, 21 reports were made, with 26 allegations of maltreatment, of which 35% were validated, all for self-neglect; 70% of self-neglect allegations reported by project social workers were validated. Results suggest that targeted case management services could reduce the incidence of self-neglect. Similar targeted interventions may help to identify, report, and address other forms of adult maltreatment.

